# Connexin expression in human acute myeloid leukemia cells: Identification of patient subsets based on protein and global gene expression profiles

**DOI:** 10.3892/ijmm.2014.2045

**Published:** 2014-12-19

**Authors:** HÅKON REIKVAM, ANITA RYNINGEN, LARS RUNE SÆTERDAL, INA NEPSTAD, BRYNJAR FOSS, ØYSTEIN BRUSERUD

**Affiliations:** 1 Institute of Clinical Science, University of Bergen, Bergen, Norway; 2 Section for Hematology, Department of Medicine, Haukeland University Hospital, Bergen, Norway; 3 Department of Health Studies, University of Stavanger, Stravanger, Norway; 4 Department of Biomedical Laboratory Sciences and Chemical Engineering, Bergen University College, Bergen, Norway

**Keywords:** connexins, acute myeloid leukemia, cluster of differentiation molecules, gene expression profiling

## Abstract

Bone marrow stromal cells support both normal and malignant hematopoiesis. Τhis support is mediated through the local cytokine network and by direct cell-cell interactions mediated via adhesion molecules and the formation of gap junctions by connexins. Previous studies on connexins in human acute myeloid leukemia (AML) have mainly focused on the investigation of leukemia cell lines. In the present study, we therefore investigated the expression of various connexins at the protein (i.e., cell surface expression) and mRNA level in primary human AML cells. The cell surface expression of the connexins, Cx26, Cx32, Cx37, Cx43 and Cx45, varied considerably between patients, and detectable levels were observed only for subsets of patients. On the whole, Cx43 and Cx45 showed the highest cell surface expression. Connexin expression was dependent on AML cell differentiation, but showed no association with cytogenetic abnormalities or mutations of the fms-related tyrosine kinase 3 (*FLT3*) or nucleophosmin (*NPM)‑1* genes. By contrast, only Cx45 showed a significant variation between patients at the mRNA level. A high Cx45 expression was associated with the altered regulation of the mitogen-activated protein kinase (MAPK) pathway and the release of pro-inflammatory cytokines [interleukin (IL)-17, tumor necrosis factor (TNF), interferon-γ], whereas a low Cx45 expression was associated with the altered regulation of protein functions (i.e., ligase activity, protein folding and catabolism). There was no significant correlation observed between the connexin mRNA and protein levels. Thus, differences in connexin expression can be used to subclassify AML patients. Differences in connexin cell surface expression profiles are not reflected at the mRNA level and have to be directly examined, whereas variations in Cx45 mRNA expression are associated with differences in cell signaling and the regulation of protein functions.

## Introduction

Bone marrow stromal cells (e.g., fibroblasts, osteoblasts, endothelial cells) support both normal and leukemic hematopoiesis (1). This support is mediated both via the local cytokine network and by direct cell-cell contact, including gap junctions formed by connexin molecules (2). This also seems to be true in acute myeloid leukemia (AML), an aggressive myeloid malignancy characterised by the accumulation of immature forms of leukemic blasts in the bone marrow (3). Stromal cells seem to be important both for leukemogenesis and chemosensitivity (1). This is supported by *in vitro* studies on exogenous cytokines known to be released by stromal cells (4–6) and by the *in vitro* co-culture of primary human AML cells with human fibroblasts (7), endothelial cells (8) and osteoblasts (9). Murine stromal cells (10) also have growth-enhancing and anti-apoptotic effects on primary human AML cells.

Gap junctions and connexins play an important role in AML (11), and the differentiation of the AML cell lines, HL-60 and PBL-985, is inhibited by stromal cells; this effect is possibly mediated by gap junctions (12). However, the results from studies on the effects of gap junctions on AML cell proliferation are conflicting. Firstly, gap junctions seem to exert antiproliferative effects on the HL-60 and KG1 AML cell lines (13). An antiproliferative effect has also been observed in the U937 AML cell line when increased Cx43 mRNA levels are induced by the expression of the AML1-ETO fusion gene; however, it is not known whether this antiproliferative effect is caused by the induction of Cx43 or by another effect of the fusion protein (14). If this antiproliferative effect, which is associated with increased Cx43 expression, is caused by Cx43 itself and not by another effect of the AML-ETO fusion protein, this may be associated with human AML, as increased Cx43 gap junction expression has also been detected in human AML bone marrow biopsies (15). By contrast, an *in vitro* study demonstrated a higher proliferative capacity of the OCIM2 AML cells with increased Cx43 expression compared with cells showing a considerably lower Cx43 expression (16). Finally, antileukemic chemotherapy has been shown to reduce Cx43 expression in normal hematopoietic cells as bone marrow levels are reduced following intensive chemotherapy compared with both AML marrow and marrow from healthy controls; however, it remains unknown as to whether chemotherapy has a similar effect on Cx43 expression in primary AML cells and whether this is a determining factor for the antileukemic effectiveness of the treatment (17). Taken together, these observations suggest that gap junctions and connexins are involved in leukemogenesis and are important for chemosensitivity. However, many of these studies are mainly based on the use of AML cell lines. Thus, in the present study, we investigated the expression of connexins in well-characterised primary human AML cells derived from unselected patients.

## Materials and methods

### Patients and cell preparation

This study was approved by the local ethics committee (Regional Ethics Committee III, University of Bergen, Bergen, Norway), and the samples were collected after obtaining written informed consent from all participants. AML blasts were derived from consecutive and thereby unselected patients with high peripheral blood blast counts (18). This selection of patients together with the analysis of fms-related tyrosine kinase 3 (*FLT3*) and nucleophosmin (*NPM*)-*1* mutations has been described previously (18,19). The expression of molecular markers for myeloid differentiation was analysed by flow cytometry, as previously described (20). We investigated connexin protein expression in 38 patients (referred to as cohort 1) and gene expression profiling (GEP) in a second group of 48 patients (cohort 2). This cohort was comparable with cohort 1 and included consecutive patients with relatively high peripheral blood blast counts. The clinical and biological characteristics of the two cohorts are presented in Table I.

### Preparation of AML cells

The leukemic cells were isolated by density gradient separation (Ficoll-Hypaque; specific density, 1.077; Nycomed Pharma, Oslo, Norway), and were stored frozen in liquid nitrogen (4,6,7,21). Leukemia peripheral blood mononuclear cells were collected from the AML patients with >95% of leukemic blasts among the mononuclear cells.

### Flow cytometry

Flow cytometric analysis to assess cell surface molecular expression was carried out as described in our previous study (7). Briefly, 1×10^6^ AML cells/ml were washed once in binding buffer [phosphate buffered saline (PBS) with 0.2% bovine serum albumin (BSA)] prior to incubation in the dark for 20 min at room temperature with FITC-conjugated antibodies against Cx26 (sc-7261), Cx32 (sc-7258), Cx37 (sc-27712), Cx43 (sc-13558) or Cx45 (sc-374354; Santa Cruz Biotechnology, Inc., CA, USA). The samples were thereafter washed once before flow cytometric analysis. For each measurement, 10,000 events were collected using a standard FACSCalibur flow cytometer (Becton Dickinson Immunocytometry Systems, San Jose, CA, USA) equipped with an Argon laser (488 nm) and a red diode laser (635 nm). A cytogram based on the forward angle light scatter (FSC) versus the right angle side scatter (SSC) was used to eliminate aggregates, debris and dead cells before fluorescence was detected. In the analysis of connexin membrane expression, only green fluorescence (FL1) was detected through the 530/30 nm band-pass filter. The fluorescence measurements were collected in the logarithmic mode. Both data from the positive cell region diagrams and the mean fluorescence intensity (MFI) were analysed using FCS Express version 3 (De Novo Software, Los Angeles, CA, USA). The positive cell regions were defined for each individual patient based on the FSC/SSC diagrams and contained <2% and >1% of the cells of the unstained negative controls.

### Microarray analysis

Global gene expression analyses were performed for a second patient cohort (Table I). We then used the Illumina iScan Reader that is based on the fluorescence detection of biotin-labelled cRNA. Total RNA (300 ng) from each sample was reverse transcribed, amplified and biotin-16-UTP-labelled, using the Illumina TotalPrep RNA Amplification kit (Applied Biosystems/Ambion, Foster City, CA, USA). The amount and quality of the biotin-labelled cRNA was controlled both by NanoDrop spectrophotometer and Agilent 2100 Bioanalyzer. Biotin-labelled cRNA 750 ng was hybridised to the HumanHT-12 v4 Expression BeadChip according to manufacturer’s instructions. The HumanHT-12 v4 BeadChip targets 47,231 probes derived primarily from genes in the NCBI RefSeq database (Release 38).

### Bioinformatics and statistical analyses

The AML cells were regarded as positive for cell surface expression when >20% of the cells stained positive for the marker by flow cytometry, as previously described (7). The statistical calculation for the correlation and regression (R^2^) analyses was performed using the Statistical Package for the Social Sciences (SPSS), version 15. Correlation analyses were performed using Spearman’s rank correlation coefficient (ϱ), and differences were regarded as statistically significant with a value of p<0.05. For comparisons between different groups, the non-parametric Mann-Whitney U test was used. Microarray mRNA levels are presented as the relative expression (22). Other bioinformatics analyses were performed using the J-Express 2012 analysis suite (MolMine AS, Hafrsfjord, Norway), as previously described (23). Hierarchal clustering analysis was performed using the Euclidean distance formula with complete linkage. Data are presented as heatmaps and genograms for each individual analysis. Gene set enrichment analysis (GSEA) was used to search for related genes that follow the same trends in the data set. A two-class unpaired data analysis using 1,000 permutations was performed, and gene ontology (GO) terms were ranked according to the enrichment score (ES).

## Results

### Primary human AML cells differ in their cell surface connexin expression

**Figure 1:**
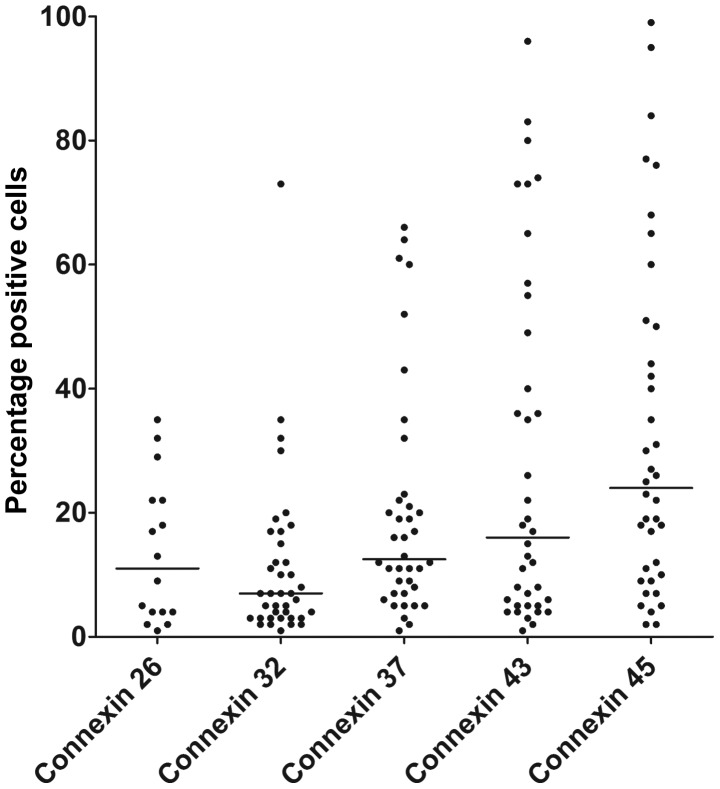
Flow cytometric analysis of cell surface connexin levels in primary human acute myeloid leukemia (AML) cells. The AML cell levels of Cx32, Cx37, Cx43 and Cx45 were determined for 38 unselected patients and the Cx26 levels for 16 unselected patients. The results are presented as the percentage of positive cells for each individual patient compared with the corresponding negative unstained control. The positive cell regions were defined for each individual patient based on the forward angle light scatter (FSC)/right angle side scatter (SSC) diagrams and contained <2% and >1% of the cells of the unstained negative control. Each dot represents a single patient, and the median level is indicated by a vertical line.

The surface membrane expression of Cx32, Cx37, Cx43 and Cx45 was analysed for primary human AML blasts derived from 38 consecutive/unselected patients; Cx26 was only analysed for 16 unselected patients. The results from flow cytometry were analysed both with regard to the percentages of positively stained cells and the MFI. The expression of all the connexins examined varied between patients (Fig. 1). The highest surface expression was observed for Cx45; 55% of the patients (21/38) had at least 20% Cx45-positive cells, whereas the fraction of patients showing at least 20% positive cells was lower for Cx43 (16/38, 42%), Cx37 (13/38, 34%), Cx32 (5/38, 13%) and Cx26 (5/16, 31%). The MFI values for Cx32, Cx37, Cx43 and Cx45 showed statistically significant correlations with each other (all p<0.001, Table II). The strongest correlation was observed between Cx43 and Cx45 expression (Spearman’s correlation, ϱ=0.930), and a strong linear correlation was also suggested by regression analysis (R^2^=0.896).

### Connexin cell surface expression is associated with the expression of myeloid differentiation markers (CD11c, CD13, CD14, CD15), but not with the expression of the CD34 stem cell marker

The correlations between the cell surface expression of connexins (Cx32, Cx37, Cx43, Cx45) and the myeloid differentiation markers, CD11c, CD13, CD14, CD15 and CD33, as well as the stem cell marker, CD34, were then analysed (Table III). All four connexins showed inverse correlations with CD13 expression (p<0.05 for all), but only Cx43 and Cx45 showed additional positive correlations with CD14 and CD15 expression (p<0.05). Furthermore, Cx45 expression positively correlated with CD11c expression (p=0.037), whereas none of the connexins showed significant correlations with CD33 or CD34 expression (Table III).

### Connexin expression correlates with the morphological signs of differentiation, but not with cytogenetic abnormalities, and FLT3 and NPM-1 mutations

**Figure 2:**
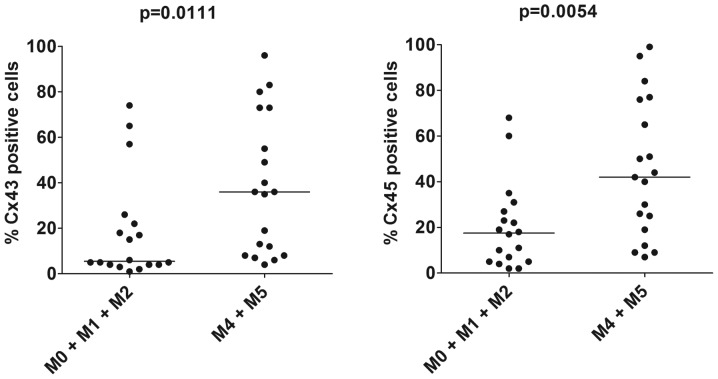
Cx43 (left panel) and Cx45 (right panel) expression by primary human acute myeloid leukemia (AML) cells: a comparison of different FAB subsets. The AML cell levels of Cx43 and Cx45 were determined for 38 unselected patients and the figure compares the levels for patients with i) AML cells showing no or minimal sign of differentiation (M0, M1 and M2), and ii) with sign of monocytic differentiation (M4 and M5). The results are presented as the percentage of positively stained cells. The expression of both Cx43 and Cx45 was significantly higher in the M4/M5 AML than in the M0/M1/M2 AML subset (p=0.0111 and p=0.0054, respectively, Mann-Whitney U test for both Cx43 and Cx45).

All patients analysed for connexin cell surface expression were classified according to the FAB criteria and this morphological/histochemical classification was used to group the patients into two major subsets as follows: i) AML cells showing no or minimal signs of differentiation (FAB classification M0, M1 and M2); and ii) signs of monocytic differentiation (FAB M4 and M5) (Fig. 2). The expression of Cx32 and Cx35 did not differ between the two subsets (data not shown), whereas Cx43 and Cx45 expression was significantly increased in the AML cells with monocytic differentiation (Fig. 2; p=0.0111 and p=0.0054, respectively, Mann-Whitney U test). There were no differences observed in connexin expression when comparing patients with different cytogenetic abnormalities and patients with or without *FLT3/NPM‑1* mutations (data not shown).

### Hierarchical clustering identifies a subset of patients with a generally low cell surface connexin expression

**Figure 3:**
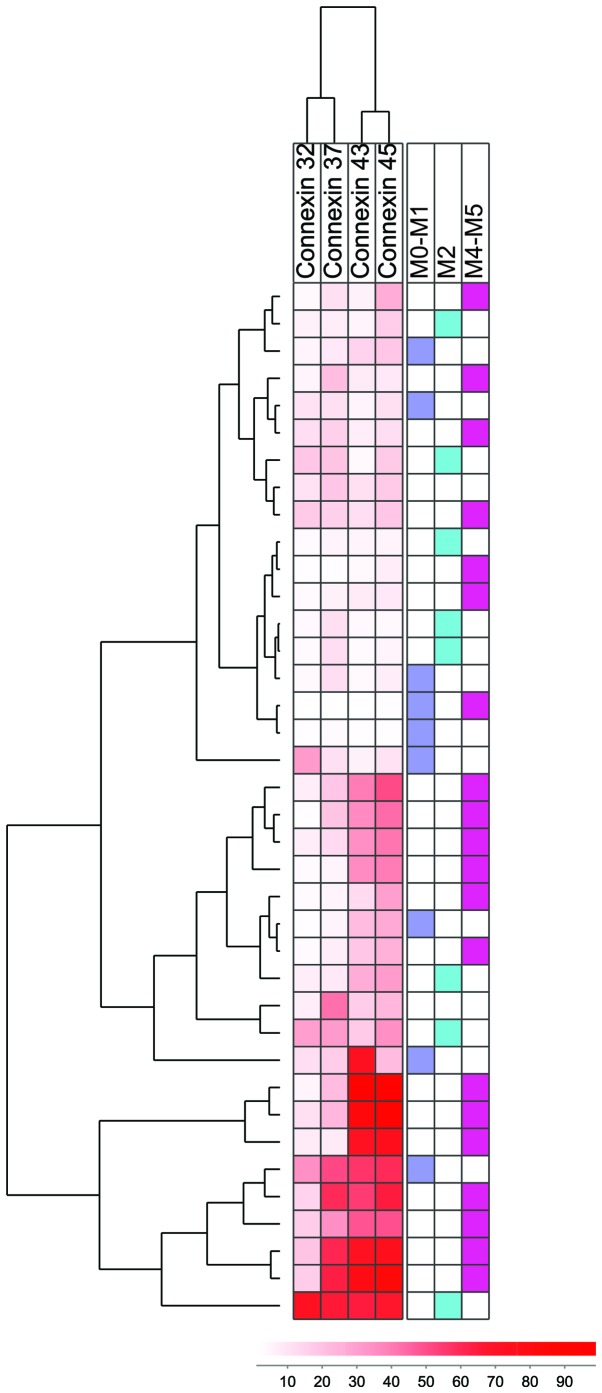
Hierarchical clustering analysis based on the expression of Cx32, Cx37, Cx43 and Cx45 in acute myeloid leukemia (AML) cells. Connexin expression was analysed by flow cytometry in 38 patients, and the analysis is based on the percentage of positively stained cells for each connexin and patient. An unsupervised hierarchical clustering was performed using the complete linkage method and Euclidean distance. The results are presented as denograms with corresponding heat maps. The darker red color indicates higher expression. The data obtained in the clustering are compared with the FAB classification of the patients (see right part of the figure).

An unsupervised hierarchal clustering was performed based on the expression of Cx32, Cx37, Cx43 and Cx45 in AML cells. This analysis was based on the percentages of positively stained cells, and the use of the complete linkage method and the Euclidean distance (Fig. 3). Cx32 and Cx37 were clustered together, and Cx43 was clustered with Cx45. The patients were divided into three main subsets based on their connexin expression: i) the first cluster consisted of 18 patients with a generally low expression of all connexins (upper section of heatmap); ii) the second cluster (11 patients, middle section of heatmap) showed intermediate levels of Cx43 and Cx45; and iii) the third cluster (9 patients, lower section of heatmap) showed generally higher connexin levels, particularly for Cx43 and Cx45. The three clusters did not differ with regard to cytogenetic abnormalities, *FLT3* or *NPM-1* mutations (data not shown), and no significant differences were observed in FAB classification between the clusters either (Fig. 3).

**Figure 4:**
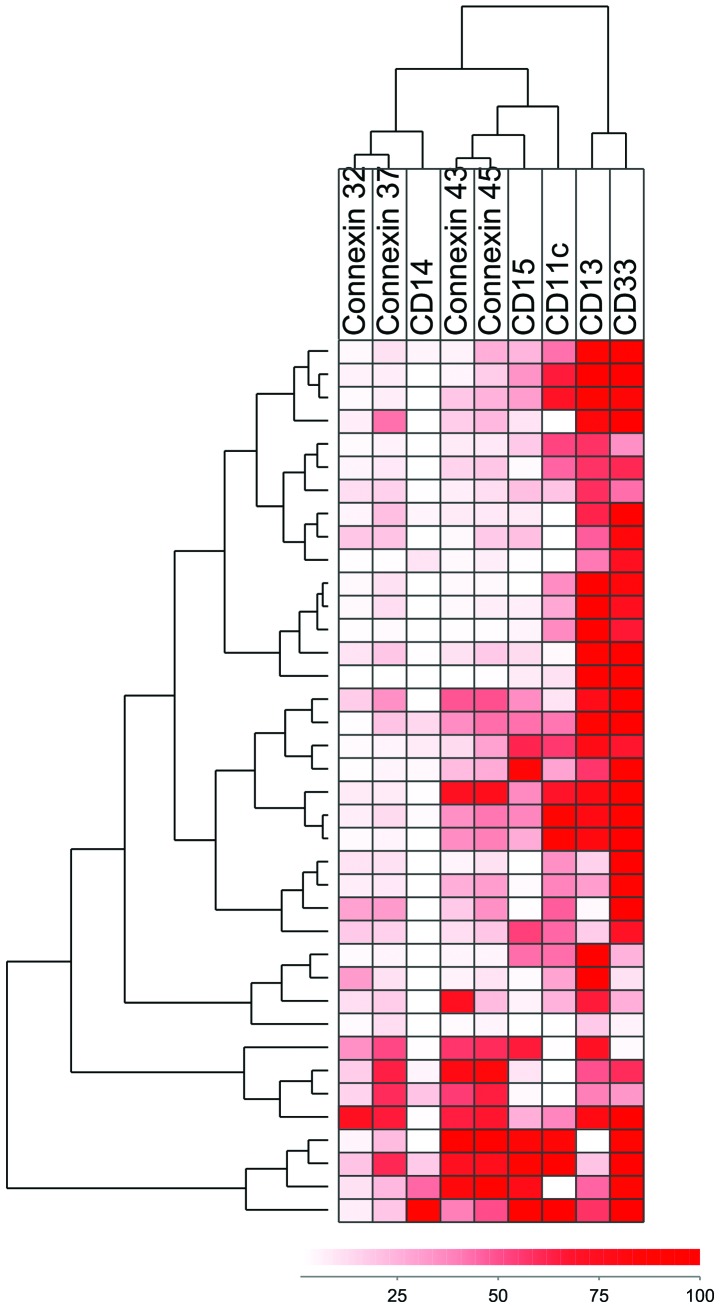
Hierarchical clustering based on the expression of connexins and molecular differentiation markers in leukemia cells. The analysis was based on the results from flow cytometric analysis presented as the percentage of positively stained cells for Cx32, Cx37, Cx43 and Cx45 in addition to the differentiation markers, CD11c, CD13, CD14, CD15 and CD33. We performed an unsupervised hierarchical clustering using the complete linkage method and Euclidean distance. The darker red color indicates higher expression.

We performed an additional hierarchical clustering analysis (complete linkage method and Euclidean distance) based on the expression of connexins and the cell surface differentiation markers, CD11c, CD13, CD14, CD15 and CD33 (Fig. 4). The two pairs of connexins (Cx32/Cx37 and Cx43/Cx45) clustered closely together in this analysis as well; CD14 clustered close to Cx32/Cx37, whereas CD15 clustered close to Cx43/Cx45. The majority of patients with a high Cx43/Cx45 expression formed a separate cluster (8 patients, lower section of heatmap).

### Only Cx45 expression shows a wide variation between patients at the mRNA level and differences in Cx45 mRNA levels are associated with distinct gene expression profiles

We used the microarray data obtained from a second cohort of AML patients (cohort 2, Table I). This cohort was comparable with cohort 1 and included consecutive patients with relatively high peripheral blood blast counts (see Materials and methods) (Table I). Among the connexins investigated, Cx45 (encoded by the *GJC1* gene) was the only one that showed a relatively high expression and a wide variation in expression between patients, whereas the other connexins showed relatively low mRNA levels and only minor variation in expression between patients. Thus, the relatively wide variation in expression between patients with regard to the connexin protein expression profile is not reflected in the mRNA expression profile, an observation suggesting that transcriptional regulation is important for the expression of several connexins.

**Figure 5:**
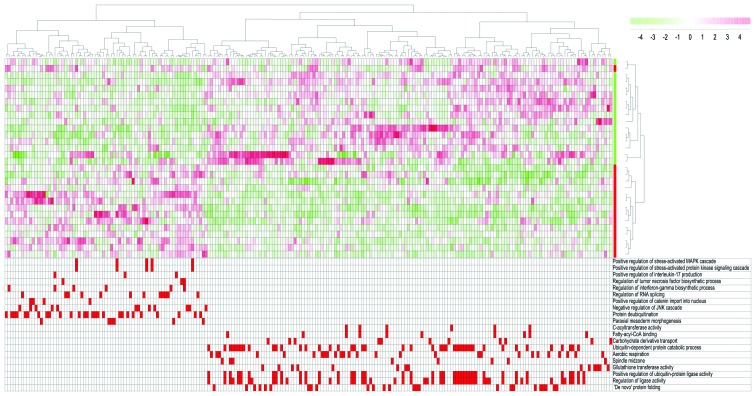
Hierarchical clustering based on gene set enrichment analysis for patients with a high Cx45 mRNA expression. We used global gene expression analyses of the acute myeloid leukemia (AML) cells for 48 consecutive patients (cohort 2), and we compared the 15 patients with highest *GJC1* expression (red column bar) and the 15 patients with the lowest *GJC1* expression (green column bar). We performed a gene set enrichment analysis (GSEA), comparing the two distinct groups. By using the leading genes belonging to the top ten gene ontology (GO) terms enriched in both the low and high groups, we were able to 225 genes enriched in at least one GO term. These genes were used to perform a hierarchical clustering analysis, dividing the two groups in two distinct clusters, except one outlier. The heatmap shows downregulated genes (green) and upregulated genes (red); the GO terms in which the marked genes belonged to are in the bottom of the figure.

We then used gene set enrichment analysis (GSEA) to compare the global gene expression profiles for two contrasting patient subsets; the first subset included 15 AML patients with the highest *GJC1* expression (*GJC1* high) and the second included 15 patients with the lowest *GJC1* expression (*GJC1* low). Using the leading genes belonging to the top ten gene ontology (GO) terms enriched in both the low and the high group, we identified 225 genes that were enriched in at least one GO term (Fig. 5). We subsequently performed a hierarchical clustering analysis based on these 225 genes; the 30 patients were then divided into two distinct subsets corresponding to the original *GJC1* high/low subsets except for one outlier. The main GO terms enriched in the *GJC1* high group included annotations related to the mitogen-activated protein kinase (MAPK) pathway and the synthesis of pro-inflammatory cytokines, interleukin (IL)-17, tumor necrosis factor (TNF) and interferon-γ. By contrast, the enriched GO terms for the *GJC1* low patients included terms related to the regulation of ligase activity, protein folding and protein catabolism. Thus, differences between AML patients with regard to Cx45 expression at the mRNA level in leukemia cells are associated with additional differences with regard to intra- and extracellular signaling, as well as the regulation of protein function.

Combined data of mRNA and protein expression was available for 19 overlapping patients between cohorts 1 and 2; however, no significant correlations were detected between the mRNA and protein levels for any connexin (data not shown). Thus, patient subclassification based on connexin expression at the protein and mRNA level identifies different subsets, and this is possibly due to post-transcriptional regulation of the protein levels.

## Discussion

Leukemic hematopoiesis is supported by various stromal cells (1). In human AML, this support seems to be caused both by communication through gap junctions formed by connexins (2), as well as adhesion molecule binding and the stromal release of leukemia-supporting cytokines (7–10). This support may be important both for AML development and chemosensitivity (11). The supportive role of gap junctions/connexins has mainly been studied in AML cell lines. In the present study, we therefore investigated connexin expression in primary human AML cells. Cx43 and Cx45 in particular showed a relatively high cell-surface expression compared with the other connexins examined. These differences between connexins may be caused by several mechanisms, including differences in mRNA expression, post-transcriptional regulation or protein turnover/degradation (24).

Animal studies have indicated that the knockout of Cx32 in bone marrow facilitates leukemogenesis (25). The low mRNA levels of Cx32 detected in the present study suggest that low Cx32 expression levels contribute to leukemogenesis in human AML, even though 13% of the AML patients showed detectable expression levles of Cx32 on the cell surface. The role of Cx32 in leukemogenesis may therefore differ and contribute to the heterogeneity in leukemogenesis between patients (26).

This study demonstrated that all investigated connexins (i.e., Cx26, Cx32, Cx37, Cx43 and Cx45) were expressed in AML cells, but detectable expression for >40% of the patients was observed only for Cx43 (42%) and Cx45 (55%), even though these two connexins showed the lowest mRNA expression. Furthermore, studies on both normal and leukemic bone marrow cells have demonstrated the formation of Cx43 gap junctions between stromal and hematopoietic cells, as well as the upregulation of Cx43 gap junctions in AML marrow (15). Cx43 gap junctions also mediate the communication between normal CD34^+^ hematopoietic stem cells and stromal cells (27); animal models with the loss of a single Cx43 allele have shown decreased normal hematopoiesis (28), and gene deletion of Cx43 in mice has been shown to decrease hematological recovery following treatment with 5-fluorouracil (29). The present study suggests that Cx43 is also important in leukemic hematopoiesis at least for a subset ofpatients.

The protein expression of several connexins showed a significant inverse (CD13) or positive correlation (CD11c, CD14, CD15) with molecular differentiation markers, and increased levels were also associated with morphological signs of differentiation. By contrast, connexin protein expression showed no association with cytogenetic abnormalities or mutations of the *FLT3* or *NPM-1* genes. Taken together, these observations suggest that differentiation is important for connexin expresssion, whereas *NPM-1* or *FLT3* mutations only have a minor impact on connexin expression.

Both Cx45 and Cx43 have been linked to stem cell functions and are expressed by human, as well as mouse embryonic stem cells (30–32). In murine cells, they can form heterodimeric gap junction channels (32). Our experiments revealed that Cx43 and Cx45 expression was closely correlated (Fig. 3); however, based on our data, it cannot be concluded whether this correlation represents a molecular basis for the formation of heterodimers.

We observed several significant correlations between the expression of various connexins (Fig. 2), even though the protein expression of Cx26 and Cx32 was very low or absent in the majority of patients, and several patients were negative for Cx37 expression. It is not certain whether Cx37 plays a role in normal or neoplastic hematopoiesis, although its expression has been detected in bone marrow endothelial cells (15), where it is important for monocyte adhesion (33). Cx37 expression may indirectly affect leukemic hematopoiesis through its involvement in AML-associated angiogenesis or interactions with AML cells in the endothelial stem cell niches.

The present study demonstrates that several connexins, Cx43 and Cx45 in particular, are expressed on the surface of primary human AML cells in a large subset of patients. The profile of connexin protein expression can be used for the subclassification of patients. This variation in protein expression cannot be detected at the mRNA level, where only Cx45 expression shows a considerable variation between patients. This Cx45 mRNA variation is also associated with differences in intracellular and extracellular cytokine signaling, as well as the regulation of protein function. These observations suggest that different subsets of patients are identified when the subclassification is based on the protein and mRNA expression of connexins.

## Figures and Tables

**Table I:** Clinical and biological characteristics of AML patients analysed for connexin expression at the protein (cohort 1) and mRNA level (cohort 2).

Patient characteristics (consecutive patients in both cohorts)	Cohort 1 (cell surface expression)	Cohort 2 (gene expression profiles)
Demographic data and disease history		
Gender (no.)		
Male/female	17/21	23/25
Age		
Median (range)	63.4 (29–88)	60.5 (24–84)
History		
*De novo*	24 (63%)	40 (83%)
Secondary	8 (21%)	4 (8%)
Relapse	6 (16%)	4 (8%)
AML cell differentiation		
FAB classification		
M0–1	9 (24%)	16 (33%)
M2	9 (24%)	10 (21%)
M3	1 (3%)	0 (0%)
M4–5	19 (50%)	22 (46%)
CD34 expression		
0–20%	16 (42%)	15 (36%)
20–50%	5 (13%)	5 (12%)
50–100%	17 (45%)	22 (52%)
Genetic abnormalities		
Cytogenetics		
Favourable	3 (9%)	2 (4%)
Intermediate	2 (6%)	2 (4%)
Adverse	8 (24%)	10 (21%)
Normal	10 (30%)	25 (52%)
Not available	10 (30%)	9 (19%)
*FLT3* mutations	15 (44%)	21 (44%)
*NPM‑1* mutations	12 (32%)	17 (35%)

AML, acute myeloid leukemia.

**Table II:** Significant correlations between the cell surface expression of different connexions in primary human AML cells.

	Cx32	Cx37	Cx43
Cx37	<0.001 (0.674)	–	Not significant
Cx43	<0.001 (0.544)	<0.001 (0.722)	–
Cx45	<0.001 (0.566)	<0.001 (0.745)	<0.001 (0.930)

The expression was analysed by flow cytometry for the 38 patients in cohort 1. Spearman’s correlation coefficient was used for the statistical analyses, and the results are presented as the p-value with the corresponding correlation coefficients (ϱ) given in parenthesis. AML, acute myeloid leukemia.

**Table III:** Correlations between the expression of different connexions and molecular differentiation markers in primary human AML cells.

	Cx32	Cx37	Cx43	Cx45
CD11c	0.388 (−0.166)	0.504 (−0.129)	0.128 (0.290)	**0.037 (0.389)**
CD13	**0.046 (−0.326)**	**0.024 (−0.366)**	**0.033 (−0.347)**	**0.020 (−0.376)**
CD14	0.984 (0.003)	0.103 (0.277)	**0.030 (0.362)**	**0.005 (0.457)**
CD15	0.938 (0.013)	0.364 (0.154)	**0.003 (0.482)**	**0.001 (0.542)**
CD33	0.951 (0.010)	0.431 (0.132)	0.408 (0.138)	0.130 (0.250)
CD34	0.258 (−0.188)	0.498 (−0.133)	0.437 (−0.130)	0.180 (−0.222)

The expression was analysed by flow cytometry for the 38 patients in cohort 1. Spearman’s correlation test was used for the statistical analysis, and the results are presented as the p-value with the corresponding correlation coefficients (ϱ) given in parentheses. Correlations with p<0.05 are marked in bold. AML, acute myeloid leukemia.
